# Knockdown of neurokinin-1 receptor expression by small interfering RNA prevents the development of allergic rhinitis in rats

**DOI:** 10.1007/s00011-013-0649-5

**Published:** 2013-08-11

**Authors:** Hong Wang, Ruxin Zhang, Jing Wu, Hua Hu

**Affiliations:** Department of Otorhinolaryngology, Huadong Hospital, Shanghai Medical College, Fudan University, Shanghai, 200040 China

**Keywords:** Allergic rhinitis, NK-1R, siRNA, Rats

## Abstract

**Objective and design:**

This study is aimed at exploring the role of neurokinin-1 receptor (NK-1R) in the development of allergic rhinitis (AR) in rats.

**Methods:**

Sprague–Dawley rats were sensitized and challenged with ovalbumin to induce AR. The rats were treated intranasally with saline, control, or NK-1R-specific small interfering RNA (siRNA) before and during the challenge period. The numbers of sneezes and nose rubs and amount of nasal secretion in individual rats were measured. The levels of NK-1R expression in the nasal mucosal tissues after the last challenge were determined. The numbers of eosinophils in the collected nasal lavage fluid and the levels of serum interleukin (IL)-5 in individual rats were determined.

**Results:**

The levels of NK-1R expression in the nasal mucosal tissues of the AR rats that had been treated with saline or control siRNA were significantly higher than those in the healthy controls and the rats treated with NK-1R-specific siRNA, demonstrating NK-1R silencing. Furthermore, knockdown of NK-1R expression significantly reduced the amounts of sneezing, nose rubbing, and nasal secretions in AR rats. Knockdown of NK-1R expression also significantly eliminated eosinophil infiltration in the nasal tissues and reduced the levels of serum IL-5 in rats.

**Conclusions:**

Knockdown of NK-1R expression decreased allergic inflammation in nasal mucosal tissues and alleviated the allergic rhinitis symptoms, suggesting that NK-1R may be a critical mediator of the development of AR.

## Introduction

Allergic rhinitis (AR) is a global health problem, which is associated with severe consequences and disabilities. AR can negatively affect life quality and school and work performance in patients [1, 2]. Currently, the precise pathogenic process of AR is not fully understood. Accumulating evidence has shown that neuro-immune abnormalities play important roles in the development of AR [3]. Substance P (SP) is a neuropeptide present in the upper respiratory tract around the blood vessels and glands, and between the muscle fibers and epithelial cells. Indeed, high levels of SP have been detected in the nasal mucosal tissues of patients with AR [4]. Previous studies have shown that SP has a wide variety of biologic functions, such as increasing vascular permeability, stimulating mucous secretion, and chemotaxis of eosinophils, which are associated with symptoms in patients with AR [5, 6]. Furthermore, exogenous SP induces nasal obstruction in a dose-dependent manner in humans and animals [7]. Intranasal treatment with capsaicin to deplete the presynaptic SP can ameliorate the AR-related symptoms. Hence, modulation of SP activity will be a promising strategy for the intervention of AR.

SP exerts its biological actions by binding to a high-affinity G-protein-coupled receptor denoted NK-1R, which is expressed by most inflammatory cells, such as mast cells and macrophages [8]. NK-1R is also expressed on epithelial cells, vascular endothelial cells, and glandular cells of the nasal mucous membranes, in combination with SP along the sensory nerves [9]. A previous study has shown that induction of AR up-regulates NK-1R mRNA transcription in the nasal mucosa of rats [10]. However, little is known about the impact of NK-1R silencing on clinical symptoms and nasal inflammation.

In the present study, we employed a rat model of AR to test how knockdown of NK-1R expression by nasal treatment with NK-1R-specific siRNA could affect clinical symptoms and nasal mucosal inflammation in vivo.

## Materials and methods

### Animals and AR model

Thirty-two male Sprague–Dawley rats weighing 210–240 g were purchased from Chinese Academy of Sciences, Shanghai, China. The animals were housed in a pathogen-free facility with free access to food and water. The experimental protocols were approved by the Animal Care and Research Committee of Fudan University.

The animal model of AR was induced as described previously [11]. Briefly, the rats were sensitized intraperitoneally with 0.2 mg ovalbumin (OVA) and 0.2 mg calmogastrin in 1 ml saline for 7 days. The rats were subjected to airway sensitization with 0.25 % OVA in 15 ml of saline by ultra-nebulization using a nebulizer (Dobio Biology Technology, Shanghai, China) for five consecutive days. One week later, the rats were challenged intranasally with 100 µl of 1 % OVA in saline 50 µl per nostril daily for 7 days. The control group (NS) of rats was administered saline alone.

The AR groups of rats were randomized and treated intranasally with 100 µl of NK-1R-siRNA (GenePharma, Shanghai, China) (the NKR group) or control NC-siRNA (GenePharma) (the NCS group) (1OD260 in 100 µl) on the day before challenge and days 3 and 6 post-challenge. The NK-1R-siRNA sequences were sense 5′-CCUACAUCAACCCAGAUCUUU-3′ and antisense 5′-AGAUCUGGGUUGAUGUAGGUU-3′, and the NC-siRNA sequences were sense 5′-UUCUCCGAACGUGUCACGUdTdT-3′ and antisense 5′-ACGUGACACGUUCGGAGAAdTdT-3′. The saline control AR group (NSAR) and normal group were treated with saline. Blood specimens were obtained from the tail vein of individual rats. The rats were killed 1.5 h after the last challenge, and their nasal mucosal tissues were sampled. One portion of the nasal mucosal sample was immediately frozen in liquid nitrogen. The remaining samples were fixed in 4 % paraformaldehyde for immunohistochemistry analysis.

### Observation

The numbers of sneezes and nose rubs were counted for 15 min immediately after the last nasal challenge. A sneeze in rats was characterized by an explosive expiration just after deep inspiration. A nose rub in rats was characterized by an external peri-nasal scratch by their forelimbs. The nasal secretions were collected as described previously [12]. One filter-paper strip was inserted into one nostril for 5 min and weighed as the amount of nasal secretion.

### Immunohistochemistry

The paraffined nasal mucosal tissue sections (4 µm) were deparaffined, rehydrated, and subjected to antigen retrieval. After being blocked with 3 % peroxide-methanol at room temperature for 10 min and with 2 % bovine serum albumin (BSA), the sections were incubated overnight with rabbit polyclonal antibodies against NK-1R (1:100 dilutions, Thermo Scientific, Rockford, IL, USA). Subsequently, the sections were exposed to biotinylated anti-rabbit and horseradish peroxidase (HRP) conjugated avidin, followed by development with 3,3-diaminobenzidine (DAB). The sections were counterstained with hematoxylin. The control sections were not exposed to the primary antibodies.

### Real time RT-PCR

Total RNA was extracted from the nasal mucosal tissues using Trizol reagent (Invitrogen, Carlsbad, CA, USA), following the manufacturer’s protocols, and reverse transcribed into cDNA using a cDNA synthesis kit (Promega, Madison, WI, USA). The relative levels of NK-1R mRNA transcripts to control β-actin were determined by quantitative RT-PCR on an ABI 7300 (Applied Biosystems, Foster City, CA, USA) using a SYBR Green-based real-time RT-PCR kit and specific primers. The sequences of primers were sense 5′-TCTTCTTCCTCCTGCCCTACA-3′, antisense 5′-CATTTCCAGCCCCTCATAATC-3′ for NK-1R (212 bp); and sense 5′-CCTCTATGCCAACACAGT-3′, antisense 5′-AGCCACCAATCCACACAG-3′ for β-actin (155 bp). The relative levels of NK-1R mRNA to β-actin were determined by the 2^−ΔΔ*Ct*^ method.

### Assessment of eosinophil infiltration

The nasal lavage fluid (NLF) was collected 1 h after the last challenge [13]. Briefly, individual rats were anesthetized and subjected to tracheotomy. Subsequently, a plastic hose was inserted through the incision of the trachea to the nasopharynx and the nasal cavities of individual rats were washed with pre-warmed saline through the tracheal hose using a micro pump (300 µl/min for 10 min). The NFL samples were collected from the anterior nares and centrifuged at 2,000*g* for 10 min. The cell pellets were re-suspended by 0.5 ml of Hanks’ solution, and the cells were stained with ethanol-eosin. The number of eosinophils in individual samples was counted using a standard hemocytometer.

### IL-5 and NK1-R quantification

Individual serum samples were prepared from individual rats by centrifugation at 1,000*g* for 10 min and stored at −80 °C until use. The concentrations of serum interleukin (IL)-5 in the different groups of rats were determined by ELISA using an IL-5 detection kit, according to the manufacturer’s instruction (RapidBio Lab, West Hills, CA, USA). The detection limitation of IL-5 was 10 pg/ml.

The frozen nasal mucosa samples were homogenized, and total proteins were extracted using cell lysis solution (Pierce, Rockford, IL, USA). After quantification of proteins, the concentrations of NK-1R in the nasal mucosa samples were determined by ELISA using the NK-1R-specific ELISA kit (GBD, San Diego, CA, USA) and expressed as µg NK-1R per mg total proteins.

### Statistical analyses

All data were expressed as the mean ± SD. The differences between groups were analyzed by analysis of variance (ANOVA) followed by post-hoc Bonferroni test using SPSS v11.5 software. A probability value of less than 0.05 was considered statistically significant.

## Results

### Knockdown of NK-1R expression by specific siRNA in the nasal mucosa of AR rats

**Fig. 1:**
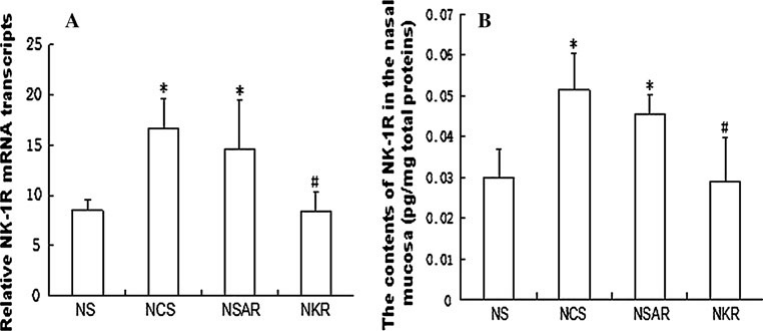
Measurement of NK-1R expression in the nasal mucosal tissues of rats. After induction of AR and treatment with control or NK-1R-specific siRNA, the different groups of rats were killed and the levels of NK-1R expression in the nasal mucosal tissues 1.5 h after the last challenge were determined by quantitative RT-PCR (**a**) and ELISA (**b**). Data are expressed as the mean ± SD of individual groups (*n* = 8 per group) of rats from three separate experiments. *NS* Healthy control rats treated with saline, *NCS* AR rats treated with control siRNA, *NSAR* AR rats treated with saline, *NKR* AR rats treated with NK-1R-specific siRNA. *P <0.05 vs. NS group. ^#^ *P* <0.05 vs. NCS or NSAR group

**Fig. 2:**
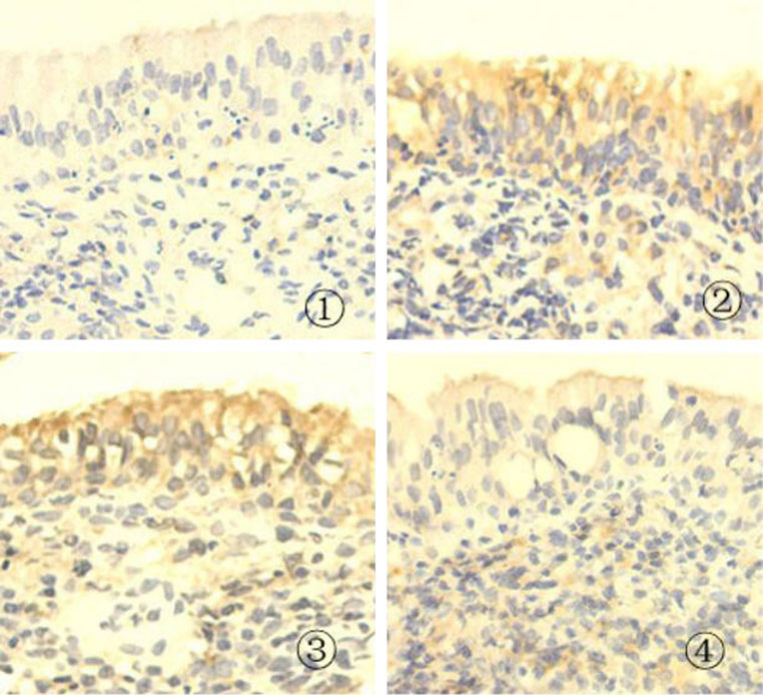
Immunohistochemistry analysis of NK-1R expression in the nasal mucosa. The nasal mucosal tissue sections from different groups of rats were stained with hematoxylin and eosin and imaged. Representative images (magnification ×400) from individual groups are shown. ① Healthy control rats treated with saline, ② AR rats treated with control siRNA, ③ AR rats treated with saline, ④ AR rats treated with NK-1R-specific siRNA

To study the role of NK-1R in AR rats, we measured the levels of NK-1R expression in the nasal mucosal samples of the different groups of rats by quantitative RT-PCR, immunohistochemistry, and ELISA. As shown in Fig. 1, while moderate levels of NK-1R mRNA transcripts were detected in the healthy controls (NS), significantly higher levels of NK-1R mRNA transcripts were found in the saline vehicle-injected AR (NCS) and NC-siRNA-injected (NSAR) rats (NCS 16.68 ± 3, NSAR 14.53 ± 4.94 vs. NS 8.5 ± 1.08, *p* < 0.05). Treatment with NK-1R-specfic siRNA significantly reduced the relative levels of NK-1R mRNA transcripts by nearly 50 % in the nasal mucosal samples (NKR 8.35 ± 2 vs. NCS 16.68 ± 3, NSAR 14.53 ± 4.94, *p* < 0.05, Fig. 1a). A similar pattern of NK-1R proteins was observed in the different groups of rats, as determined by ELISA (NS 0.0298 ± 0.0069, NCS 0.0515 ± 0.0091, NSAR 0.0455 ± 0.0047, NKR 0.0291 ± 0.0108, *P* = 0.0001, Fig. 1b). Further immunohistochemistry analyses revealed that NK-1R was expressed in the cytoplasm of epithelial cells in the nasal mucosa and some granules (Fig. 2). While weak anti-NK-1R staining was detected in the nasal mucosal tissues of the NS rats, a markedly increased intensity of anti-NK-1R staining was observed in the nasal mucosal tissues of the NCS and NSAR rats. However, weak anti-NK-1R staining was detected on the mucociliary layer of the nasal mucosal tissues from the NKR rats. These three parallel lines of data clearly demonstrated that induction of AR up-regulated NK-1R expression in the nasal mucosal tissues of rats and that intranasal treatment with NK-1R-specific siRNA effectively inhibited NK-1R expression in the nasal mucosal tissues of rats.

### Knockdown of NK-1R expression alleviates AR symptoms in rats

**Fig. 3:**
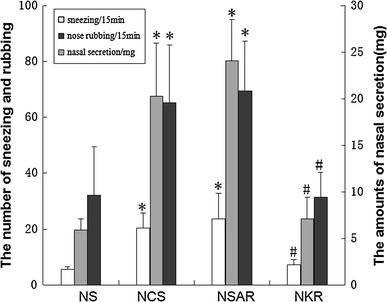
Knockdown of NK-1R expression mitigates AR-related clinical symptoms in rats. After the last challenge, the numbers of sneezes and nose rubs in individual rats were observed for 15 min, and the amount of nasal secretions was collected for 5 min. Data are expressed as the mean ± SD of individual groups (*n* = 8 per group) of rats. *NS* Healthy control rats treated with saline, *NCS* AR rats treated with control siRNA, *NSAR* AR rats treated with saline, *NKR* AR rats treated with NK-1R-specific siRNA. *P <0.05 vs. NS group. ^#^ *P* <0.05 vs. NCS or NSAR group

Animals with AR usually develop sneezing and nose rubbing symptoms and have increased amounts of nasal secretions [14]. To determine the role of NK-1R in AR, we observed clinical symptoms in the different groups of rats. Following saline challenge, the NS group of rats displayed a few sneezes and nose rubs and secreted a little mucous during the observation period (Fig. 3). In contrast, the NCS and NSAR groups of rats presented significantly increased numbers of sneezes (NCS 20.4 ± 5.3, NSAR 23.7 ± 9.2 vs. NS 5.6 ± 0.8, *p* < 0.01) and rubs (NCS 65.3 ± 20.7, NSAR 69.5 ± 17.9 vs. NS 32 ± 17.4, *p* < 0.01), accompanied by increased amounts of nasal secretion (NCS 20.3 ± 5.7, NSAR 24.1 ± 4.4 vs. NS 5.9 ± 1.2, *p* < 0.001). However, the number of sneezes (NKR 7.2 ± 1.9 vs. NCS 20.4 ± 5.3, NSAR 23.7 ± 9.2, *p* < 0.01) and rubs (NKR 31.4 ± 8.9 vs. NCS 65.3 ± 20.7, NSAR 69.5 ± 17.9, *p* < 0.01), and the amounts of secreted mucous (NKR 7.1 ±2.3 vs. NCS 20.3 ± 5.7, NSAR 24.1 ± 4.4, *p* < 0.001) in the NKR group of rats were significantly less than those of the other AR rats. Therefore, knockdown of NK-1R expression ameliorated AR-related clinical symptoms in rats.

### Knockdown of NK-1R expression inhibits eosinophil infiltration and reduces serum IL-5 levels in rats

**Fig. 4:**
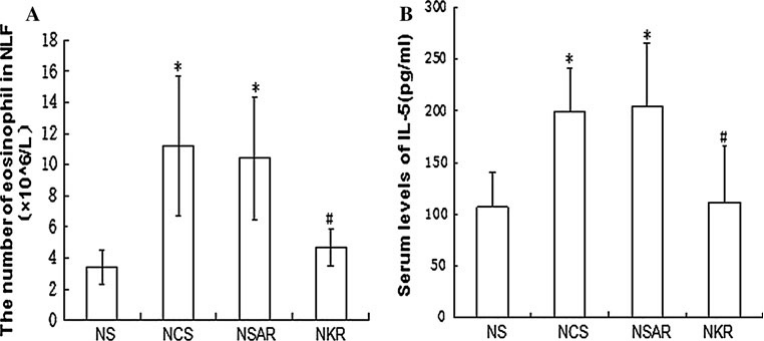
Knockdown of NK-1R expression reduces the numbers of eosinophil infiltrates in NLF and the levels of serum IL-5 in AR rats. After the last challenge, the NLF was collected from individual rats and the numbers of eosinophils in the NFL were measured (**a**). In addition, the concentrations of serum IL-5 in individual rats were determined by ELISA (**b**). Data are expressed as the mean ± SD of individual groups (*n* = 8 per group) of rats from three separate experiments. *NS* Healthy control rats treated with saline, *NCS* AR rats treated with control siRNA, *NSAR* AR rats treated with saline, *NKR* AR rats treated with NK-1R-specific siRNA. *P <0.05 vs. NS group. ^#^ *P* <0.05 vs. NCS or NSAR group

Next, we examined the impact of NK-1R silencing on allergic inflammation in the nasal mucosa. We found that the numbers of eosinophils in the NLF from the NSAR and NCS groups of rats were significantly greater than those from the NKR group (NCS 11.2 ± 2.7 × 10^6^/L, NSAR 10.4 ± 2.3 × 10^6^/L vs. NKR 4.7 ± 1.2 × 10^6^/L, *p* < 0.05), which was only slightly higher than, but not significantly different from, those in the NS group (NKR 4.7 ± 1.2 × 10^6^/L vs. NS 3.4 ± 1.1 × 10^6^/L, *p* > 0.05, Fig. 4a). A similar pattern in the levels of serum IL-5 was detected in the different groups of rats (NCS 199.3 ± 42.5, NSAR 204.6 ±61.2 vs. NKR 111.2 ± 54.3, *p* < 0.01; NKR 111.2 ±54.3 vs. NS 106.7 ± 33.7, *p* > 0.05, Fig. 4b). These data indicated that induction of AR recruited eosinophil infiltration and induced strong IL-5 responses in rats. Knockdown of NK-1R expression reduced the levels of eosinophil infiltration and IL-5 responses in rats.

## Discussion

In the present study, we found that induction of AR up-regulated NK-1R expression in the nasal mucosa of rats, which was associated with clinical symptoms and eosinophil-related nasal mucosal inflammation. Intranasal treatment with NK-1R-specific siRNA effectively inhibited NK-1R expression and mitigated AR-related clinical symptoms and eosinophil inflammation in the nasal mucosal tissues of rats. Our findings support the notion that NK-1R is involved in nasal mucosal inflammation and suggest that NK-1R may be a crucial mediator of the development of AR. Indeed, the vascular permeability induced by tachykinins in the nasal mucosa is primarily mediated by NK-1R, and it can be controlled by local application with a NK-1R antagonist [15]. NK-1/2 receptors are both involved in the development of nasal hyperresponsiveness in allergic rhinitis of guinea pigs [16]. Conceivably, modulation of NK-1R expression may be a promising strategy for the intervention of AR.

SP acts as an important factor participating in the pathogenesis of nasal mucosal inflammation, such as AR [17–19]. SP can stimulate the release of inflammatory mediators, including histamine and chemokines, which recruit inflammatory infiltrates [20, 21]. Indeed, topical administration of SP can induce AR symptoms and recruit eosinophil infiltration in the nasal mucosal tissues of healthy animals [6, 22].

Capsaicin binds to the TRPV1 receptor to cause membrane depolarization and massive SP depletion from the presynaptic nerves [23]. Repeated treatment with capsaicin can deplete SP in the nasal mucosa and relieve AR clinical symptoms [24]. SP can bind to its NK-1R, which is expressed by inflammatory cells and nasal mucosal epithelial cells as well as vascular endothelial cells [9]. We found that induction of AR significantly up-regulated NK-1R expression in the nasal mucosal tissues, which was associated with increased numbers of sneezes and nose rubs and increased amounts of mucosal secretion in rats. Our data were consistent with previous reports [9, 10]. Given that NK-1R is expressed by inflammatory cells and nasal mucosal epithelial cells as well as vascular endothelial cells [9], it is possible that the up-regulated NK-1R expression in those cells increases their sensitivity to SP, increasing nasal mucosal secretion, vasodilatation, plasma extravasation, and inflammation. Therefore, our findings and those of others strongly suggest that NK-1R is a mediator of the pathogenesis of AR in rats.

Eosinophils and IL-5 are crucial for the development of AR [25–27]. IL-5 is mainly produced from Th2 cells and/or mast cells. IL-5 can recruit eosinophil infiltration in the nasal mucosal tissue, where eosinophils can secrete cationic proteins, such as the major basic protein, peroxidase, and neurotoxin, leading to tissue damage [28]. The induction of AR increased the numbers of eosinophil infiltrates in the nasal mucosal tissues, accompanied by elevated levels of serum IL-5 in rats, further supporting the importance of eosinophil infiltration and IL-5 responses in the development of AR. SP can synergistically enhance eosinophil infiltration mediated by IL-5 and stimulate human eosinophil degranulation and the release of neurotoxin and peroxidase, which can be abolished by NK-1R antagonists [29–31]. Indeed, administration of SP enhances IL-5 mRNA transcription in nasal mucosal tissues of patients with allergic rhinitis [32]. In addition, NK-1R can regulate the activation of mast cells through FcεRI signaling [33]. Therefore, therapeutic targeting of SP or NK-1R to inhibit IL-5 expression and eosinophil infiltration may be valuable for the intervention of AR.

To further study the role of NK-1R in AR, we treated AR rats intranasally with NK-1R-specific siRNA and found that treatment with siRNA not only dramatically reduced the levels of NK-1R expression, but also relieved AR-related clinical symptoms in rats. In addition, knockdown of NK-1R significantly reduced the numbers of eosinophil infiltrates in the nasal mucosal tissues and the levels of serum IL-5 in rats. Our data were consistent with previous findings that treatment with a NK-1R antagonist inhibits allergic nasal mucosal inflammation [34]. Treatment with a dual antagonist for the NK1/2 receptors suppresses the development of nasal hyperresponsiveness, and intravenous administration of NK-1R receptor antagonist before the intranasal application of SP inhibits SP-induced nasal obstruction [16, 34]. Therefore, knockdown of NK-1R in Th2 cells may directly attenuate their function and reduce IL-5 production. Given that IL-5 is predominantly secreted by Th2 cells and mast cells, it is possible that knockdown of NK-1R may inhibit mast and other cell activation, lower IL-5 expression and reduce eosinophil infiltrates in the nasal mucosal tissue of rats. We are interested in further investigating whether knockdown of NK-1R expression can inhibit Th2 and mast cell infiltration in the nasal mucosa in AR rats.

The siRNA approach is an effective and specific technology for reducing target gene expression. A large research effort has been directed at developing potential siRNA therapeutics for the treatment of viral infections, cancer, and neurodegenerative diseases [35, 36]. Although the effect of siRNA has been demonstrated in animal models, the efficacy of siRNA-based therapeutic strategies in humans has not been clarified and is currently being tested in ongoing clinical trials [37]. In our study, we designed a specific NK-1R-siRNA and demonstrated that intranasal administration of NK-1R effectively inhibited NK-1R expression in rats. Therefore, our findings suggest that siRNA-based NK-1R-specific therapy may be feasible for the intervention of AR.

In summary, our data indicated that intranasal treatment with NK-1R-specific siRNA not only dramatically reduced the levels of NK-1R expression, but also relieved AR-related clinical symptoms and nasal mucosal inflammation in rats. Hence, our findings support the notion that SP and NK-1R are crucial for the development of AR. We recognize that our study had limitations, such as not examining different types of inflammatory cells, such as Th2, mast cells, neutrophils and macrophages in the nasal mucosa, and the lack of detailed studies of molecular mechanisms by which knockdown of MK-1R affected Th2 cell function and IL-5 production and the signal pathways involved in nasal mucosal epithelial cell function in rats. Therefore, further studies to address these questions are warranted.
